# A Comprehensive Monitoring Study on Electrocardiographic Assessments and Cardiac Events After Fingolimod First Dose—Possible Predictors of Cardiac Outcomes

**DOI:** 10.3389/fneur.2020.00818

**Published:** 2020-08-12

**Authors:** Volker Limmroth, Tjalf Ziemssen, Ingo Kleiter, Bert Wagner, Stephan Schmidt, Christoph Lassek, Monika Baier-Ebert, Guillaume Wendt, Ralf Dechend, Wilhelm Haverkamp

**Affiliations:** ^1^Department of Neurology, Cologne General Hospitals, University of Cologne, Cologne, Germany; ^2^Center of Clinical Neuroscience, University Clinic Carl Gustav Carus Dresden, Dresden, Germany; ^3^Department of Neurology, St. Josef Hospital, Ruhr-University Bochum, Bochum, Germany; ^4^Marianne-Strauß-Klinik, Behandlungszentrum Kempfenhausen für Multiple Sklerose Kranke, Berg, Germany; ^5^NeuroMVZ, Stuttgart, Germany; ^6^Neurologische Gemeinschaftspraxis Schmidt, Neudecker, Viebahn und Kronenberger, Bonn, Germany; ^7^Neurologische Gemeinschaftspraxis Kassel und Vellmar, Kassel, Germany; ^8^Novartis Pharma GmbH, Nuremberg, Germany; ^9^Experimental and Clinical Research Center, Charité-Campus Buch and HELIOS Klinikum, Berlin, Germany; ^10^Division for Metabolism and Cardiology, Department of Cardiology, Charité Universitaetsmedizin Berlin, Berlin, Germany

**Keywords:** fingolimod, first dose, electrocardiogram, cardiac effects, bradycardia, atrioventricular conduction

## Abstract

**Background:** First dose observation for cardiac effects is required for fingolimod. Previous results in patients with relapsing remitting multiple sclerosis (RRMS) suggest that transient bradycardia and conduction abnormalities during the observation phase are rare, benign and reversible. Prior analyses corroborate these findings. The present large scale dataset allows subgroup analyses for differences in the incidence of cardiac findings depending on patient characteristics.

**Methods:** START was an open-label, multi-center study that enrolled 6,998 RRMS patients. Primary endpoints were incidence of bradycardia (heart rate < 45 bpm) and second-/third-degree atrioventricular (AV) block during treatment initiation. Subgroup analyses were performed according to age, gender, body mass index (BMI), baseline expanded disability status scale (EDSS), and concomitant medication to determine the impact of these variables on cardiac outcomes parameters.

**Results:** 63 patients (0.9%) developed bradycardia (<45 bpm), 120 patients (1.7%) had a second-degree Mobitz I (Wenkebach) block and/or 2:1 AV block. One case of an asymptomatic third-degree AV block occurred. No Mobitz II AV block was observed. After 1 week, no second-/third-degree AV block was observed. The incidence of second- or third-degree AV blocks was significantly higher in older patients (≥50 years; *p* = 0.014 vs. patients 35–49 years). Second- or third-degree AV blocks were more frequent in females (87.5% of all patients with a second- or third-degree AV block; *p* < 0.001), while bradycardia occurred more often in males (58.7% of all bradycardia events; *p* < 0.001). Furthermore, patients with a BMI below 25 had a higher incidence of second- or third-degree AV block.

**Conclusions:** In summary, transient bradycardia and AV conduction abnormalities after the first dose of fingolimod were rare and asymptomatic. When compared to females, male patients might have a higher risk for bradycardia during treatment initiation, presumably due to a lower resting heart rate. Furthermore, a low heart rate before treatment initiation, low body weight, or low BMI possibly increases the risk for bradycardia. Second- or third-degree AV blocks were more frequent in females, older patients and patients with a low BMI. Nevertheless, these cardiac events remained rare and benign, confirming the favorable cardiac safety profile of fingolimod upon treatment initiation in MS patients without cardiovascular comorbidities.

## Introduction

Fingolimod (FTY720, brand name Gilenya®) has been approved for the treatment of relapsing remitting multiple sclerosis (RRMS). It exerts its therapeutic effects via modulation of sphingosine-1-phosphate (S1P-) receptors on lymphocytes (1–5). As receptors of this class, predominantly S1P1, are also expressed on atrial myocytes (6), fingolimod mediates a transient decrease in heart rate and prolongation of atrioventricular (AV) conduction that is limited to treatment initiation (7–10). The first dose of fingolimod was found to result in an increase in parasympathetic activity. It was suggested that this effect is responsible for an initial heart rate decline (11, 12) and measures of parasympathetic activity could be a predictive marker of first dose events (13, 14). Furthermore, cardiac autonomic tone at baseline was found to predict the magnitude of heart rate decrease (15).

In the pivotal trials, first-degree AV blocks (prolonged PR interval) following drug initiation were detected in 4.7% of patients on fingolimod 0.5 mg and 1.7% of patients on placebo. Second-degree AV block was detected in 0.2% patients on fingolimod 0.5 mg. The conduction abnormalities were typically transient, asymptomatic, and resolved within 24 h on treatment. START was a prospective study that aimed to assess the cardiac safety of fingolimod first dose in detail in a much larger cohort compared to previous studies in daily practice treatment circumstances (14, 16–22). The study followed the EU label first-dose observation recommendation by obtaining a Holter ECG for 6 h in addition to clinical monitoring and pre-/post first dose 12-lead ECG. Previously published results of an interim analysis with 3,951 patients have shown that clinically relevant cardiac events did not correspond with abnormalities on continuous ECG monitoring and conduction abnormalities as demonstrated by Holter ECG usually remained clinically asymptomatic (23). The results suggest that continuous Holter ECG monitoring after fingolimod first dose likely may not add clinically relevant value to patient safety with respect to bradycardia or conduction abnormalities in a general RRMS population without cardiovascular comorbidities.

This final analysis was conducted after enrolment of another 3,000 patients, resulting in 6.998 enrolled patients. With its far larger and unique sample size it was aimed to substantiate the findings of the interim analyses and to investigate the impact of patient or disease characteristics like age, gender, BMI, EDSS status, or concomitant medication on the cardiac outcomes parameters studied.

## Patients and Methods

### Study Design, Approvals, and Participants

START (NCT01585298; www.clinicaltrials.gov) was an open-label, multi-center study in Germany in patients with RRMS receiving fingolimod at a daily dose of 0.5 mg. Its purpose was (a) to assess cardiac safety of first dose fingolimod with a special focus on bradycardia and AV conduction abnormalities, and (b) to analyze individual arrhythmic episodes more extensively in order to evaluate the clinical relevance of continuous ECG monitoring. The study protocol was approved by the respective state and institutional ethical standards committees at all participating sites (Competent ethics committee: Ethikkommission der Ärztekammer Nordrhein; EUDRACT No. 2012-000653-32-DE). Patients had to give written informed consent. The inclusion and exclusion criteria have been published previously (23). Patients were allowed to repeatedly participate in the START study in case they had previously discontinued fingolimod.

Between June 2012 and December 2016, 6,998 patients were enrolled in 278 centers in Germany, of which 6,961 (99.5%) completed the study. Sample size calculation was revised based on the observed incidence of 0.963% of a second-degree or higher AV block in the first 1,000 patients enrolled, which was lower than originally anticipated. With a sample size of 7,000, a two-sided 95% confidence interval for a single proportion extended 0.0023 or 0.23% from the observed proportion for an expected proportion of 0.00963.

### Endpoints

Primary endpoints were the incidence of bradycardia (heart rate <45 bpm) as measured by pulse palpation by study personnel, and the incidence conduction block (second-degree AV block type Mobitz I (Wenkebach block), second-degree AV block type Mobitz II second degree, 2:1 AV block, and third-degree AV block) during the 6 h Holter monitoring period. Secondary endpoints included the incidence of other conduction abnormalities such as first-degree AV block (PR-interval prolongation), and QTc prolongation [Fridericia correction of the QT intervals: QTc = QT/third root [RR]].

Furthermore, the incidence of adverse events (AE), serious adverse events (SAE), AE suggestive for cardiac origin, and the incidence of overnight hospitalization was determined. The occurrence of any cardiac symptoms was assessed in patients with an identified second- or higher degree AV block. Furthermore, continuous ECG recordings of patients with an AV block were evaluated rigorously. The duration of pauses (RR intervals) and the minimal heart rate during a second-degree AV block were determined *post hoc* by the cardiologist. Holter ECG data of patients with cardiac symptoms were assessed for any abnormalities that might have predicted the symptoms. Details on the endpoint definitions have been published previously.

### Subgroup Analyses

Subgroup analyses were performed according to age, gender, BMI, baseline EDSS, use of concomitant medication known to prolong the QT/QTc interval, and use of disease-modifying treatment to determine the impact of these variables on cardiac outcome parameters (incidence of bradycardia and incidence of AV block second-degree or higher). Subgroup analyses according to gender were also performed for the incidence of QTc interval prolongation.

### Study Procedures

Patients were assessed at screening, pre-dose, post-dose, and end-of-study (after 7 days). Prior to the first dose of fingolimod a 12-lead standard ECG was recorded. First dose monitoring consisted of continous Holter monitoring for 6 h. At day 7 another 12-lead ECG was recorded. The study procedures were described in detail in an earlier publication.

### Statistics

Results of START were analyzed using a logistic regression model, see Supplemental Tables 1, 2 for details. Where indicated, additional statistical tests were performed using Student's *t*-test or Fisher's exact test.

## Results

### Study Patients and Conduct

**Figure 1:**
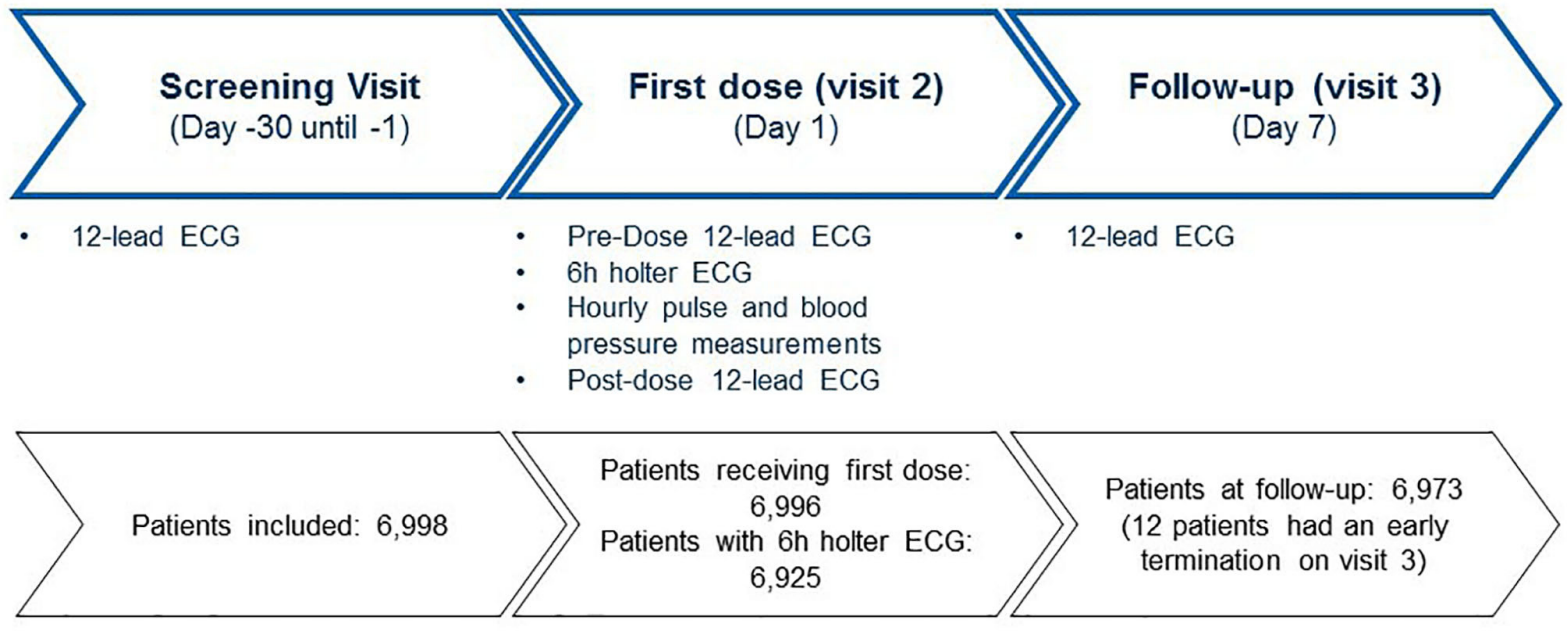
Patient disposition.

The majority of the patients were seen by office-based neurologists. Baseline demographics and MS history are presented in Table 1. A Holter ECG was recorded at visit 2 in 6,925 (99%) out of 6,996 patients who received fingolimod. Two patients of the 6,998 enrolled persons did not receive the study drug (Figure 1).

**Table 1:** Patient demographics and relevant baseline characteristics.

	**Enrolled patients (*N* = 6,998)**
**Age**	
Mean ± SD	39.2 ± 10.5
**Age groups in years**, ***n*** **(%)**	
18–34	2,504 (35.8)
35–49	3,234 (46.2)
> = 50	1,260 (18.0)
**Sex**, ***n*** **(%)**	
Female	4,924 (70.4)
Male	2,074 (29.6)
**Body mass index (kg/m**^2^**)**	
Mean ± SD (*n* = 6,965)	25.4 ± 5.2
Missing	33 (0.5)
<18.5	244 (3.5)
18.5–24.9	3,630 (51.8)
25–29.9	1,945 (27.8)
≤30	1,146 (16.4)
**Relevant comorbidities**	
History of diabetes, *n* (%)	123 (1.8)
History of hypertension, *n* (%)	803 (11.7)
**EDSS**	
Mean ± SD (*n* = 6,960)	2.7 ± 1.6
Missing	38 (0.5)
0–3.5	5,331 (76.2)
> 3.5–6	1,370 (19.6)
> 6	259 (3.7)
**DMT treatment within the last 6 months**, ***n*** **(%)**	
No	1,474 (21.1)
Yes	5,524 (78.9)

### Changes in Heart Rate

On average, patients receiving the first dose of fingolimod experienced a transient decline in heart rate. 89.5% of the patients reached the nadir before the end of the 6 h post-dose observation period (mean 3.7 h; SD 1.16). In 63 patients (0.9%) heart rate dropped below 45 bpm (95%-CI: 0.68–1.12) during treatment initiation. The average pre-dose heart rate of these patients was 58.8 ± 9.10 bpm. At visit 3, bradycardia was observed in 5 patients (0.07%) (see Table 2). Table 3 presents the heart beat dynamics during treatment initiation by time of the lowest heart rate measurement, by presence of bradycardia and by presence of AV block.

**Table 2:** Incidence of bradycardia.

**Fingolimod 0.5 mg; *n* = 6,996**	**Baseline predose**	**Baseline post dose**	**7 days post dose**
No bradycardia, *n* (%)	6,991 (99.99%)	6,933 (99.10%)	6,974 (99.93%)
Bradycardia with HR <45 bpm, *n* (%)	1 (0.01%)	63 (0.90%)	5 (0.07%)

**Table 3:** Heart rate dynamics by subgroup within 6 h post-dose.

	**Number of patients *n* (%)**	**Pre-dose heart rate^**a**^ (bpm) mean (range)**	**Lowest post-dose heart rate^**a**^ (bpm) mean (range)**	**Maximum decline in heart rate^**a**^ (bpm) mean (SD)**
	**Fingolimod**, ***N*** **=** 6,985*			
**Overall population**				
Overall population	6,985* (100.0)	74.1 (42–132)	66.2 (40–120)	11.9 (8.52)
**By time of lowest heart rate**				
Patients with lowest heart rate at <6 h	6,265 (89.7)	74.0 (42–132)	66.0 (40–120)	12.0 (8.46)
Patients with lowest heart rate at 6 h	720 (10.3)	74.5 (47–120)	63.6 (38–90)	10.9 (8.94)
**By presence of bradycardia**				
Patients with bradycardia	63 (0.9)	58.8 (42–91)	46.6 (31–68)	16.7 (9.36)
**By presence of second-degree AV block**				
Patients with second-degree AV block	120 (1.7)	73.9 (53–100)	65.2 (46–93)	12.9 (8.69)

a *As measured by on-site study personnel*.

* *Only patients with values at baseline pre-dose and post-dose are included*.

**Figure 2:**
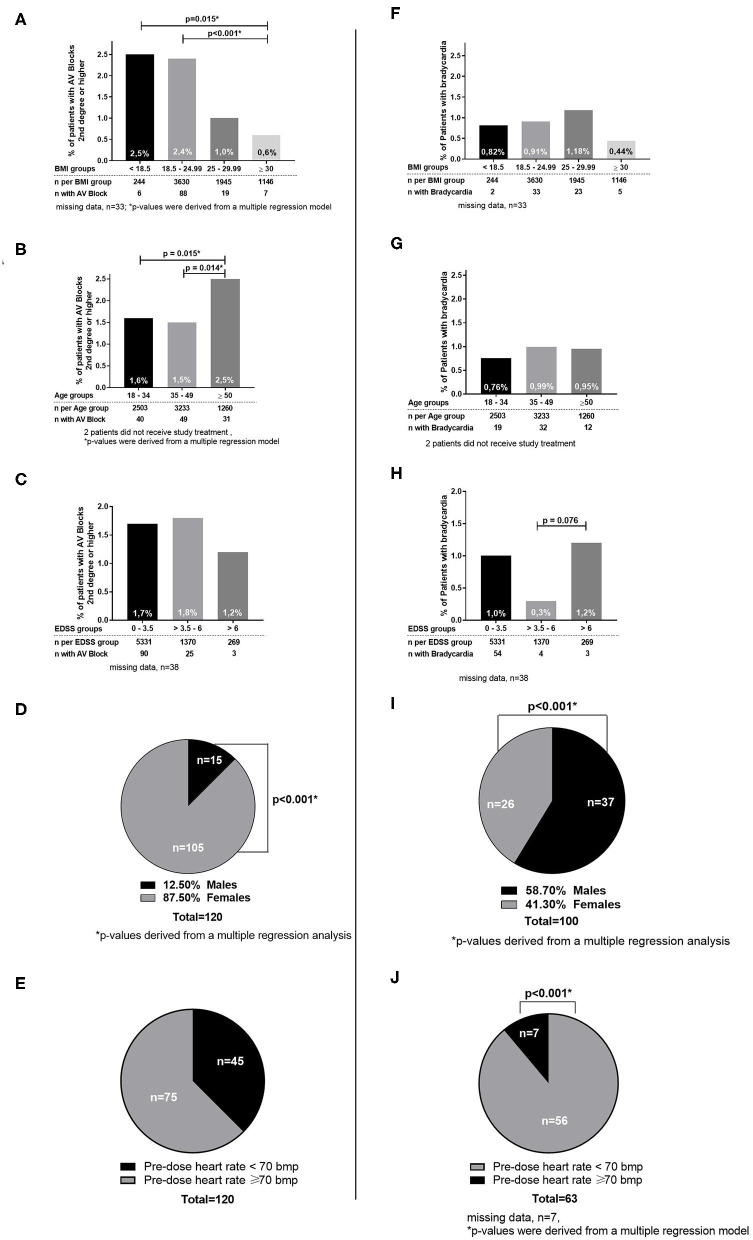
Cardiac outcomes by subgroups. **(A–E)** Subgroups for patients with AV Blocks 2nd degree or higher. **(F–J)** Subgroups for patients with bradycardia.

Male patients had a significantly higher incidence of bradycardia (1.78%) than females (0.53%; *p* < 0.001; Figure 2). These events occurred primarily during the first 5 h. Of note, baseline heart rates in female patients were lower than those in male patients (72.5 vs. 74.7; *p* < 0.0001) and incidence of bradycardia was significantly higher in patients with a pre-dose heart rate below 70 bpm. The maximum decline in heart rate was not different between males and females (*p* = 0.9094).

Patients with an EDSS at baseline over 6 had a significantly higher incidence of bradycardia (1.2%) vs. patients with an EDSS between 3.5 and 6 (0.3%; *p* = 0.076; Figure 2).

The incidence of bradycardia was lowest in the subgroup of patients with a BMI of ≥30. Furthermore, higher weight was significantly correlated with a higher minimum heart rate in the total population (*p* < 0.01). Analysis by age subgroups revealed no differences in the incidence of bradycardia in any age category. Subgroup analysis of patients with vs. without concomitant medication known to prolong QT intervals (e.g., SSRI, tricyclic antidepressants, amantadine) (see Table 4), of patients with vs. without prior disease-modifying therapy (e.g., beta-interferons, glatiramer acetate, natalizumab) showed no significant differences for bradycardia or AV block (data not shown), which is in line with previous studies regarding SSRIs (24).

**Table 4:** Patient characteristics with potential relevance for cardiac events and frequency of symptoms by subgroup.

	**Overall population**	**Patients with bradycardia**	**Patients with second-degree AV block or higher**
	***N* = 6,998^**a**^**	***N* = 63^**b, c**^**	***N* = 120^**c**^**
**Demographics**			
Age (years), mean ± SD	39.2 ± 10.52	40.1 ± 10.33	40.6 ± 12.02
Female, *n* (%)	4,924 (70.36)	26 (41.27)	105 (87.50)
**Concomitant medication known to prolong QT interval**			
SSRI *n* (%)	707 (10.10)	1 (1.59)	8 (6.67)
TCA *n* (%)	160 (2.29)	1 (1.59)	2 (1.67)
Amantadine *n* (%)	69 (0.99)	0	0
Carbamazepine	49 (0.70)	0	2 (1.67)
Fampridine *n* (%)	523 (7.47)	4 (6.35)	8 (6.67)
**Potassium levels at visit 2**			
<3.5 mmol/L	32 (0.46)	1 (1.61)	0
≥ 3.5–5.5 mmol/L	6,778 (97.83)	61 (98.39)	119 (99.17)
> 5.5 mmol/L	118 (1.70)	0	1 (0.83)
**Cardiac symptoms**^**d**^ **during 6 h first-dose observation**			
Patients with symptoms, *n* (%)	205 (2.93)	4 (6.35)^e^	2 (1.67)^f^

a *Two patients did not receive fingolimod. Information of potassium levels was missing for 70 patients*.

b *Information of potassium levels was missing for one patient*.

c *One of the patients had both, bradycardia and second-degree AV block and is therefore included in both groups*.

d *Cardiac symptoms are defined as the following MedDRA preferred terms: Angina pectoris, chest discomfort, dizziness, dyspnoea, dyspnoe exertional, fatigue, palpitations, syncope, vertigo, vertigo positional, blurred vision*.

e *Fatigue, dizziness, chest discomfort, vertigo*.

f *Palpitations and dizziness*.

### AV Conduction Abnormalities

Slowed AV conduction (i.e., first-degree AV block: PR-interval >200 ms) was observed in 124 patients (1.8%) before and 308 patients (4.4%) after the first dose of fingolimod. At visit 3, the incidence of a first-degree AV block was identical to that before treatment initiation (1.8% of patients) (see Table 5).

**Table 5:** Incidence of AV block during the course of the study.

**Fingolimod 0.5 mg; *n* = 6,996**	**Baseline predose**	**Baseline post dose**	**7 days post dose**
Patients with first-degree AV block, *n* (%)	124 (1.8)	308 (4.4)	123 (1.8)
Patients with second-degree AV block or higher, *n* (%)	0 (0.00)	120 (1.70)	0 (0.00)
Mobitz type I (wenckebach)^a^	0 (0.00)	117 (1.69)	0 (0.00)
AV Block 2:1^a^	0 (0.00)	42 (0.61)	0 (0.00)
Mobitz type II (mobitz)	0 (0.00)	0 (0.00)	0 (0.00)
Patients with third-degree AV block, *n* (%)	0 (0.00)	1 (0.01)	0 (0.00)

a *A patient might experience both Mobitz type I and 2:1 AV block during the 6-h monitoring*.

In 120 patients, a second-degree AV block was observed after treatment initiation. Of these, 117 patients (1.67%) had a Mobitz type I second-degree AV block. 43 type 2:1 blocks were identified in 42 patients (0.61%), with 39 patients having both a Mobitz type I and a 2:1 block within the observation period, and three patients exhibiting a 2:1 AV block only. No episode of clear AV block type Mobitz II was observed. An episode of a short lasting third-degree AV block was detected in a 55 years old woman 3 h 38 min after the first dose of fingolimod. The duration of this third-degree AV block was 4,750 ms. No clinical symptoms were observed. The patient was monitored overnight and fingolimod treatment was withdrawn. No AV block was observed the day after the first dose of fingolimod in this patient. At visit 3, 12-lead ECG did not reveal any second- or third-degree AV block in any patient.

Of the 120 patients with a second- or third-degree AV block during treatment initiation, 105 were females (2.10% of all females) and 15 males (0.7%; *p* < 0.001). The highest incidence (2.5%) was observed in patients with a very low BMI (<18.5) and the lowest incidence (0.6%) in patients with a high BMI (≥30; *p* = 0.015; Figure 2).

Considering all second- or third-degree AV blocks, patients between the age of 18 and 49 had a significantly lower incidence (1.6/1.5%) compared to patients ≥50 years (2.5%; *p* = 0.015/0.014). No significant difference in the incidence of AV block (second- or third-degree) was observed in the different EDSS subgroups (Figure 2) or between patient groups with pre-dose heart rate below and over 70 bpm. Subgroup analysis of patients with vs. without concomitant medication known to prolong QT intervals, of patients with vs. without disease-modifying therapy, and START repeaters vs. non-repeaters showed no significant differences by group (data not shown).

**Figure 3:**
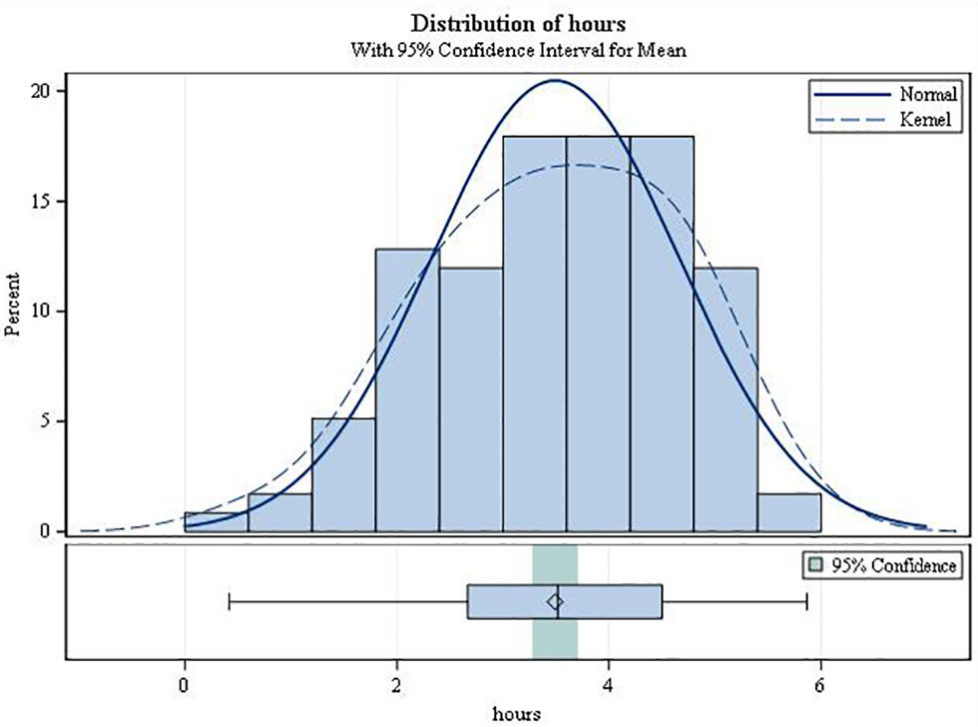
Time of the first second-degree AV block (type Mobitz I or 2:1 AV block).

**Figure 4:**
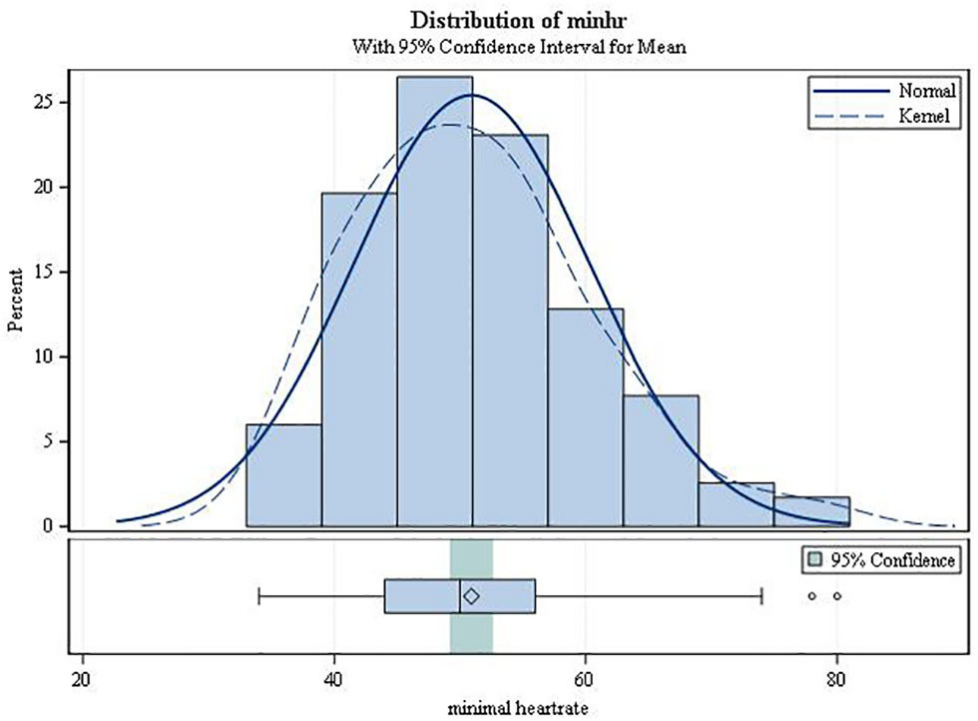
Minimum heart rate from ECG during second-degree AV block (type Mobitz I or 2:1 AV block).

**Figure 5:**
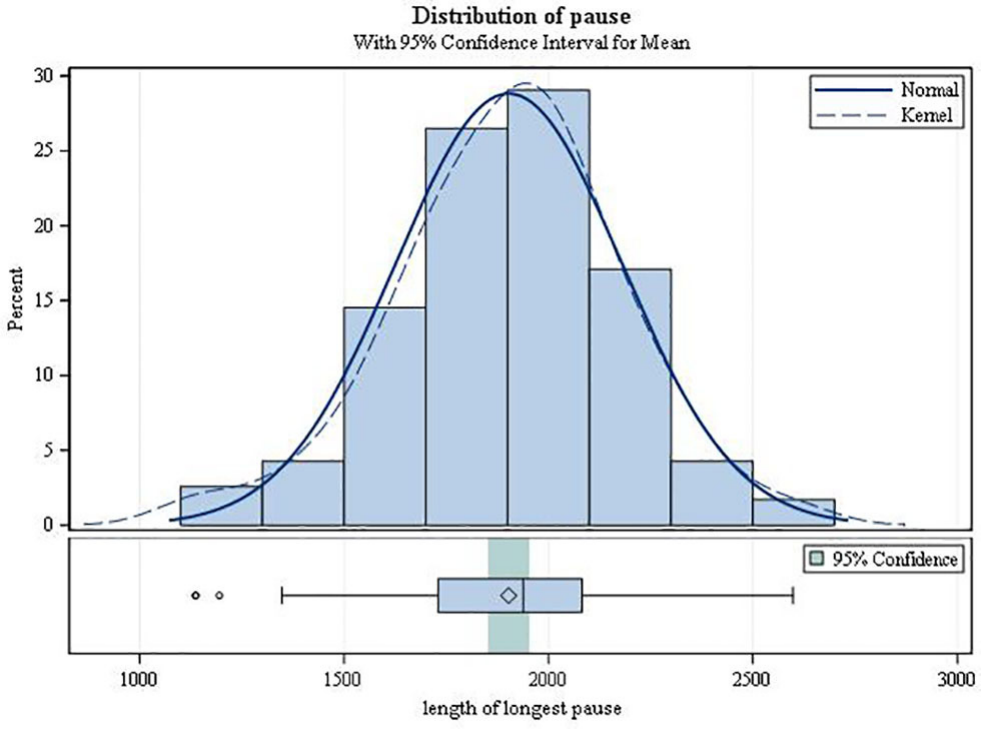
Duration of the longest pause during second-degree AV block (type Mobitz I or 2:1 AV block).

On average, the second-degree AV block became manifest 3.49 h after drug intake (SD 1.17; range 0.42–5.87; Figure 3). It always resolved spontaneously. In female patients, the mean time to first occurrence was 3.61 h (SD = 1.12), while in males it was 2.68 h (SD = 1.20) (*p* = 0.0034). The mean (SD) minimal heart rate during second-degree AV block as assessed by Holter ECG (mean of 10 consecutive beats) was 50.91 ± 9.42 bpm (range 34–80 bpm; Figure 4) with no significant difference between males and females (*p* = 0.6955). The minimum heart rate of patients with second-degree AV block as measured by study personnel was 58.76 for males and 61.14 for females (*p* = 0.2682). The longest pause observed in association with second-degree AV block was 2,598 ms, the upper limit of the 95%-CI was below 2,000 ms. The mean value of the longest pause was 1,902.6 (SD 276.8; Figure 5) with no significant difference between males and females (*p* = 0.1526).

### Effects on Repolarization

Applying >450 ms in men and >470 ms in women as a conservative threshold, only six men and seven women had QTc intervals above their respective thresholds after fingolimod intake. All of these patients continued with the study medication. No patient had a QTc interval exceeding 500 ms, which would have warranted overnight monitoring as defined in the current EU label.

A linear trend with age could be observed for prolonged QTc intervals, as defined above. In the age group 18–34 the incidence was 0.10%, in the age group 35–49 years 0.5%, and in the oldest population (50 years and older) 0.7% (*p* = 0.0022). Pairwise comparisons showed that the incidence in the youngest age group compared with the two older age groups was significantly lower (data not shown).

At visit 3, QT/QTc prolongation was observed in 13 patients, with higher incidence in males (0.39%) than in females (0.10%; thresholds for QTc were >450 and >470 ms, respectively).

### Adverse Events and Cardiac Symptoms

AEs were observed in 2.288 patients (32.7%) and 160 patients (2.3%) experienced SAEs (Table 6). No deaths occurred. The most frequent types of AEs causing discontinuation of medication were cardiac and nervous system disorders (0.4 and 0.11%, respectively). Symptoms reported as AE during the 6 h monitoring suggestive of cardiac events occurred in 205 patients (2.93%, Table 3). Females did experience significantly more cardiac adverse events, 7.41 vs. 5.98% (*p* = 0.0311).

**Table 6:** Most frequent (serious) adverse events.

	**Enrolled patients** ***N*** **=** **6,998**^**a**^	
	**Number of patients, *n* (%)**	**Number of events, *n***
**Summary of adverse events**		
Any adverse event	2.288 (32.7)	3,698
Any serious adverse event	160 (2.3)	204
Any adverse event leading to discontinuation of study drug	68 (1.0)	89
**Common adverse events (>1% by PT)**		
Headache	317 (4.53)	328
Fatigue	228 (3.26)	231
Nausea	144 (2.06%)	146
Nasopharyngitis	137 (1.96%)	137
Dizziness	129 (1.84%)	131
Atrioventricular block second degree	123 (1.76%)	151
Lymphopenia	120 (1.71%)	121
Atrioventricular block first degree	116 (1.66%)	118
Diarrhea	98 (1.40%)	98
Multiple sclerosis relapse	95 (1.36%)	96
Bradycardia	72 (1.03%)	72
Vertigo	70 (1.0%)	71
**Common serious adverse events (>0.05% by SOC/PT)**		
Cardiac disorders	80 (1.14)	99
AV block second-degree (type Mobitz I or 2:1 AV block)	49 (0.70)	60
Bradycardia (heart rate <45 bpm)	24 (0.34)	24
Nervous system disorders	41 (0.59)	45
MS relapse	26 (0.37)	26
Investigations	10 (0.14)	10
Electrocardiogram QT prolonged	4 (0.06)	4
Heart rate decreased	4 (0.06)	4
General disorders and administration site conditions	5 (0.07)	6
Infections and infestations	9 (0.13)	9
Gastrointestinal disorders	6 (0.09)	8
Vascular disorders	5 (0.07)	5
Psychiatric disorders	4 (0.06)	4

*PT, Preferred term; SOC, System organ class*.

a *Two patients did not receive fingolimod*.

Two cases of symptomatic second-degree AV block with palpitations and dizziness and four cases of symptomatic bradycardia were observed. Symptoms of bradycardia were fatigue, dizziness, chest discomfort, and vertigo. Neither syncope nor episodes of dyspnoea were observed. None of the patients with cardiac symptoms had a pause longer than 2,000 ms. No supraventricular tachycardias were observed. Four patients had more than 1,000 ventricular extra beats (VES) and another 4 patients had between 100 and 1,000 VES. 10–100 VES were observed in 22 patients and <10 VES in 174 patients. Paired VES occurred in only two patients (one couplet and one triplet). Salves or R on T phenomena were not detected. Bigeminus or trigeminus were present in 14 patients and 10 patients presented with sinustachycardia of >130 bpm.

In total, 69 patients (0.99%) were hospitalized overnight due to any cardiac adverse event that started during day 1 after first dose administration.

## Discussion

This final analysis of the START study demonstrates that only 0.9% of the patients developed bradycardia after treatment initiation. Symptomatic bradycardia was observed in 0.06%. Symptoms were always mild and transient (without the need of any treatment). Episodes of second-degree AV block (Mobitz Type I and 2:1) were detected in 1.70% of the patients and a third-degree AV block in one patient. Despite the large size of the cohort and the transient occurrence of second-degree AV block Type I and 2:1, no Mobitz II and only one third-degree AV block, which was asymptomatic and reversible, were observed. At visit 3, no patient had a second- or third-degree AV block. The observed incidences of AV block are consistent with those reported from previous studies, including pivotal and post-marketing studies. Accordingly, in these other studies, bradycardia occurred in up to 1.4%, Mobitz type I second-degree AV block (Wenckebach) in up to 2.6% and 2:1 second-degree AV block in up to 1.4% of the patients (6–10).

The events identified by continuous ECG monitoring in the present study were neither associated with severe clinical symptoms nor with clinically relevant pauses or clinically relevant drops in heart rate. The time of the initial occurrence of the second-degree AV block corresponds to the time of the heart rate nadir. Furthermore, patients with clinical symptoms had no pathological findings on Holter ECG. The results of the final analysis further support the assumption that continuous Holter ECG monitoring does not add clinically relevant value to patients' safety.

Not surprisingly, the mean heart rate at baseline of patients who developed bradycardia after the first dosing was below 60, whereas in the total population the mean baseline heart rate was above 70. Further, the incidence of bradycardia was higher in subgroups with a lower vs. higher BMI and in males vs. females. The data on EDSS subgroups showed a significantly higher incidence of bradycardia in patients with an EDSS over 6 and a trend to a higher incidence in the subgroup with an EDSS below 3.5 compared to an EDSS of 3.5 to 6. It has to be pointed out, that the distribution of patients between subgroups was highly imbalanced with a very low number of patients in the group with EDSS higher than 6. Significant differences between groups might thus result from group size imbalances rather than from clinical effects. Our data show that EDSS does not affect the occurrence of an AV block and heart rate decline. The incidence of second-degree AV block or higher was highest in patients with a BMI below 18.5 and lowest in patients with a BMI of 30 and higher. The incidence also significantly increased with higher age, i.e., in patients 50 years or older.

According to our results, patients with a low heart rate at treatment onset may be at a higher risk to develop substantial bradycardia. The results of an analysis of the heart rate variability of 81 patients suggest that enhanced parasympathetic activity is a potential mechanism for the decline in heart rate. Baseline heart rate therefore was considered to predict the risk for bradycardia caused by fingolimod (11). In fact, the cardiovascular effects of fingolimod observed after first dosing resemble the effects of parasympathetic signaling of the autonomous nervous system (15). Atropine has been shown to antagonize the initial heart rate decrease after fingolimod dosing (25). Cardiac autonomic regulation at baseline was found to predict the magnitude of fingolimod-initiated heart rate decrease (26). Heart rate variability assessment can be used to analyze cardiac autonomic regulation (12, 14, 27). An increased variability indicates parasympathetic stimulation while a decrease indicates sympathetic stimulation. After initiation of fingolimod treatment an initial increase in heart rate variability was observed that decreases within 3 months (28), while heart rate remained lower after 3 months of treatment (29) Hilz et.al. hypothesize that the observed initial parasympathetic changes under fingolimod might be beneficial for patients (30). However, after the initial predominance of parasympathetic signaling, the sympathetic nervous system is activated. It was suggested, that this is due to the S1P1 downregulation after initial S1P1 receptor agonism (15, 31). However, these effects and their clinical relevance in patients in general and in patients with cardiac risk factors need further evaluation.

In the present study, a higher BMI was associated with a lower risk for bradycardia. Moreover, the lower the body weight was the lower were the minimal heart rates. Similar results were observed in a small cohort of retrospectively analyzed patients (32). A low BMI has previously been linked to a higher incidence of fingolimod-induced adverse events. The incidence of lymphopenia has been shown to be increased in patients with a BMI lower than 18.5. The results have to be interpreted with caution due to the low number of underweight patients (33). Nevertheless, taken together with the large scale dataset from the present study, patients with a low BMI might have a higher incidence of fingolimod-induced adverse events. However, the overall risk remains low and the observed events are benign.

Gender differences were observed for the occurrence of second- and third-degree AV block and bradycardia. Males had a higher incidence of bradycardia and females had a higher incidence of AV block, which is in line with previous studies (14). The difference was statistically significant for both outcome parameters, but the magnitude of the effect was far more pronounced with regard to the occurrence of AV blocks. The time of occurrence of an AV block differed between males and females, however no difference was observed with respect to the duration of pauses, indicating that the severity of second-degree AV block did not differ between males and females. Gender-specific cardiac differences have been observed before, which might be explained by hormone-driven regulation of ion channels (34). However, the pathophysiological basis has not been fully elucidated (34, 35). The absence of relevant pauses may contribute to the fact that the vast majority of events were not associated with any symptoms. While 120 patients had a second-degree AV block type Mobitz I, only one asymptomatic third-degree block was observed. It can be assumed that AV blocks associated with fingolimod treatment initiation are benign, self-limiting and associated with a very low propensity to progress to a complete AV block. Although affected by fingolimod, overall AV conduction remains preserved. In the present study, AV blocks were not preceded by excessive slowing of heart rate as could be anticipated, but rather followed by increases in heart rate. This finding suggests that slowing of AV conduction does not result from an increased parasympathetic tone but might more likely be due to the positive rate-dependent electrophysiological effect of fingolimod. A mild direct effect of fingolimod on the AV node was found in rodents, in which high S1P1 receptor mRNA expression was present in the AV node. Fingolimod prolonged the cycle length in isolated AV node cells by 9% from 230 to 251 ms. Under pathological conditions (such as ischemia/reperfusion), fingolimod did not prolong AV conduction any further. Therefore, fingolimod-induced AV node conduction abnormalities seem to be the result of a direct effect on ion channels expressed in the AV node with mild prolongation of AV conduction time (36).

## Conclusions

The present study is the largest study to assess the cardiac effects upon fingolimod treatment initiation. Bradycardia and AV conduction abnormalities were rare, transient, and benign. Male patients were at a higher risk for bradycardia during treatment initiation. Furthermore, a low heart rate before treatment initiation, low body weight or low BMI increased the risk for bradycardia. Second- or third-degree AV blocks were more frequent in older patients as well as in patients with low BMI. In summary, the study results suggest that fingolimod therapy is safe in an outpatient setting, without the need of continuous Holter ECG. ECG monitoring during the first 6 h after treatment initiation is sufficient to record all clinically relevant cardiac effects. In summary, the present study confirms that cardiac effects upon treatment initiation of fingolimod are rare, benign, and reversible, substantiating the favorable cardiac safety profile of fingolimod in MS patients without cardiovascular comorbidities.

## Data Availability Statement

The datasets presented in this article are not readily available because the datasets generated during and/or analyzed during the current study are available from the corresponding author on reasonable request. Requests to access the datasets should be directed to wilhelm.haverkamp@charite.de.

## Ethics Statement

The studies involving human participants were reviewed and approved by Ethikkommission der Ärztekammer Nordrhein as leading ethics committee, the study protocol was approved by the respective state and institutional ethical standards committees at all participating sites (EUDRACT No. 2012-000653-32-DE). The patients/participants provided their written informed consent to participate in this study.

## Author Contributions

VL: design and conceptualization of the study, interpretation of the data, and revising the manuscript for intellectual content. TZ, IK, BW, SS, and CL: revising the manuscript for intellectual content. MB-E: statistical expert, interpretation of the data, and revising the manuscript for intellectual content. GW, RD, and WH: interpretation of the data and revising the manuscript for intellectual content. All authors contributed to the article and approved the submitted version.

## Conflict of Interest

VL has received speaker's honoraria, financial research support, or consultancy fees from Antisence, Allergan, Allmiral, Bayer, Biogen, Bionorica, Genzyme, Novartis, Roche, Sanofi, and Teva. TZ has received compensation for consulting services from Almirall, Biogen Idec, Bayer, Genzyme, Merck, Novartis, Sanofi, Teva, and has received research support from Biogen, Novartis, Sanofi, and Teva. IK has received speaker honoraria and travel funding from Bayer, Biogen, Novartis, Merck, Sanofi Genzyme, Roche; speaker honoraria from Mylan; travel funding from the Guthy-Jackson Charitable Foundation; consulted for Alexion, Bayer, Biogen, Celgene, Chugai, IQVIA, Novartis, Merck, Roche; and received research support from Chugai, Diamed. SS has received speaker's honoraria from Bayer, Biogen, Genzyme, Merck, Novartis, Roche, Sanofi, Teva, and financial research support from Bayer and Merck. MB-E is employed by Novartis Pharma GmbH, Germany. GW is employed by Novartis Pharma GmbH, Germany. RD has received speaker's honoraria, financial research support, or consultancy fees from Berlin Chemie, Novartis, Alnylam, Boehringer Ingelheim, Bayer, MSD. WH was working as consultant to Novartis. The remaining authors declare that the research was conducted in the absence of any commercial or financial relationships that could be construed as a potential conflict of interest.

## Abbreviations

AE: Adverse event
AV: Atrioventricular conduction
BMI: Body Mass Index
bpm: Beats per minute
CI: Confidence Interval
DSMB: Data Safety Monitoring Board
ECG: Electrocardiogram
EDSS: Expanded Disability Status Scale
EU: European Union
EUDRACT: European Clinical Trials Database
FTY720: Fingolimod
HR: Heart rate
i.e.: id est
MedDRA: Medical Dictionary for Regulatory Activities
mg: milligram
mmol: millimol
ms: millisecond
MS: Multiple Sclerosis
PT: Preferred Term
RRMS: Relapsing remitting multiple sclerosis
S1P: Sphingosine-1-phosphate
SAE: Serious adverse event
SD: Standard deviation
SOC: System Organ Class
US: United States.
