# Supplementary material for: A Comprehensive Monitoring Study on Electrocardiographic Assessments and Cardiac Events After Fingolimod First Dose—Possible Predictors of Cardiac Outcomes
Source: Front Neurol. 2020 Aug 12;11:818. doi: 10.3389/fneur.2020.00818 (PMC7434833; doi:10.3389/fneur.2020.00818)
Supplement: Supplementary file 1 [file Table_1.docx]

Supplemental Table 1: Logistic regression: Patients with second or third degree AV block

|  | **Odds ratio** | **95% Wald CI for odds ratio** |
| --- | --- | --- |
| Female vs Male | 2.825 | [1.633,4.890] |
| Age: 18 - 34 vs Age: ≥ 50 | 0.534 | [0.322,0.887] |
| Age: 35 - 49 vs Age: ≥ 50 | 0.557 | [0.349,0.889] |
| BMI: 18.5 - 24.9 vs BMI: ≥ 30 | 4.199 | [1.935,9.111] |
| BMI: 25 - 29.9 vs BMI: ≥ 30 | 1.686 | [0.700,4.062] |
| BMI: < 18.5 vs BMI: ≥ 30 | 3.913 | [1.299,11.789] |
| EDSS: 0 - 3.5 vs EDSS: > 6 | 1.710 | [0.530,5.519] |
| EDSS: > 3.5 - 6 vs EDSS: > 6 | 1.630 | [0.487,5.460] |
| Pre-dose heart rate < 70 bmp vs  Pre-dose heart rate ≥ 70 bmp | 1.065 | [0.727,1.560] |

| **Analysis of Maximum Likelihood Estimates** | | |
| --- | --- | --- |
| **Parameter** | **Estimate** | **p-value** |
| Intercept | -6.02 | <.001 |
| Female | 1.04 | <.001 |
| Age: 18 - 34 | -0.63 | 0.015 |
| Age: 35 - 49 | -0.59 | 0.014 |
| BMI: 18.5 - 24.9 | 1.43 | <.001 |
| BMI: 25 - 29.9 | 0.52 | 0.244 |
| BMI: < 18.5 | 1.36 | 0.015 |
| EDSS: 0 - 3.5 | 0.54 | 0.370 |
| EDSS: > 3.5 - 6 | 0.49 | 0.428 |
| Pre-dose heart rate < 70 bmp | 0.06 | 0.748 |

Supplemental Table 2: Logistic regression: Bradycardia < 45 bpm

|  | **Odds ratio** | **95% Wald CI for odds ratio** |
| --- | --- | --- |
| Female vs Male | 0.351 | [0.207,0.594] |
| Age: 18 - 34 vs Age: ≥ 50 | 0.680 | [0.309,1.497] |
| Age: 35 - 49 vs Age: ≥ 50 | 0.973 | [0.480,1.971] |
| BMI: 18.5 - 24.9 vs BMI: ≥ 30 | 1.682 | [0.650,4.353] |
| BMI: 25 - 29.9 vs BMI: ≥ 30 | 1.753 | [0.651,4.720] |
| BMI: < 18.5 vs BMI: ≥ 30 | 2.086 | [0.394,11.036] |
| EDSS: 0 - 3.5 vs EDSS: > 6 | 0.859 | [0.258,2.859] |
| EDSS: > 3.5 - 6 vs EDSS: > 6 | 0.253 | [0.055,1.152] |
| Pre-dose heart rate < 70 bmp vs  Pre-dose heart rate ≥ 70 bmp | 12.722 | [5.764,28.078] |

| **Analysis of Maximum Likelihood Estimates** | | |
| --- | --- | --- |
| **Parameter** | **Estimate** | **p-value** |
| Intercept | -5.83 | <.001 |
| Female | -1.05 | <.001 |
| Age: 18 - 34 | -0.39 | 0.338 |
| Age: 35 - 49 | -0.03 | 0.939 |
| BMI: 18.5 - 24.9 | 0.52 | 0.284 |
| BMI: 25 - 29.9 | 0.56 | 0.267 |
| BMI: < 18.5 | 0.74 | 0.387 |
| EDSS: 0 - 3.5 | -0.15 | 0.805 |
| EDSS: > 3.5 - 6 | -1.37 | 0.076 |
| Pre-dose heart rate < 70 bmp | 2.54 | <.001 |
